# A specific role for phosphoinositide 3-kinase and AKT in osteoblasts?

**DOI:** 10.3389/fendo.2012.00088

**Published:** 2012-07-20

**Authors:** Imelda M. McGonnell, Agamemnon E. Grigoriadis, Eric W.-F. Lam, Joanna S. Price, Andrew Sunters

**Affiliations:** ^1^Department of Veterinary Basic Sciences, The Royal Veterinary College,London, UK; ^2^Department of Craniofacial Development and Stem Cell Biology, King’s College London, Guy’s Hospital,London, UK; ^3^Department of Surgery and Cancer, Imperial College London, Hammersmith Hospital,London, UK; ^4^School of Veterinary Sciences, University of Bristol,Bristol, UK

**Keywords:** osteoblast, phosphoinositide 3-kinase, PI3K, AKT, protein kinase B

## Abstract

The phosphoinositide 3-kinase and AKT (protein kinase B) signaling pathway (PI3K/AKT) plays a central role in the control of cell survival, growth, and proliferation throughout the body. With regard to bone, and particularly in osteoblasts, there is an increasing amount of evidence that the many signaling molecules exert some of their bone-specific effects in part via selectively activating some of the generic effects of the PI3K/AKT pathway in osteoblasts. There is further data demonstrating that PI3K/AKT has the capacity to specifically cross-talk with other signaling pathways and transcriptional networks controlling bone cells’ development in order to fine-tune the osteoblast phenotype. There is also evidence that perturbations in the PI3K/AKT pathway may well be responsible for certain bone pathologies. In this review, we discuss some of these findings and suggest that the PI3K/AKT pathway is a central nexus in the extensive network of extracellular signaling pathways that control the osteoblast.

## INTRODUCTION

A fine balance between the bone forming activities of osteoblasts and the bone resorptive effects of osteoclasts, substantially influenced by osteocytes (Bonewald, 2011; Long, 2012) provides the skeleton with the correct architecture and strength to support everyday loads without fracture. Osteoblasts are derived from a pool of multipotent mesenchymal stem cells (MSCs) which are able to form bone, cartilage, muscle, adipose, and connective tissue via activation of distinct differentiation programs (Long, 2012). Thus, the phenotype of a mature osteoblast reflects its molecular history in terms of signaling molecule exposure, signal transduction pathway activation, and changes engendered in transcriptional networks, as well as epigenetic modifications. It is a reflection of this *cellular context* that a panoply of seemingly ubiquitous signaling molecules is capable of producing such a uniquely specific outcome, i.e., the transition from an MSC to a mature osteoblast. In this review, we will highlight the role of one pathway, the phosphoinositide 3-kinase and AKT signaling pathway (PI3K/AKT), in osteoblast differentiation and homeostasis.

## THE PHOSPHOINOSITIDE 3-KINASE AND AKT SIGNALING PATHWAY

**FIGURE 1:**
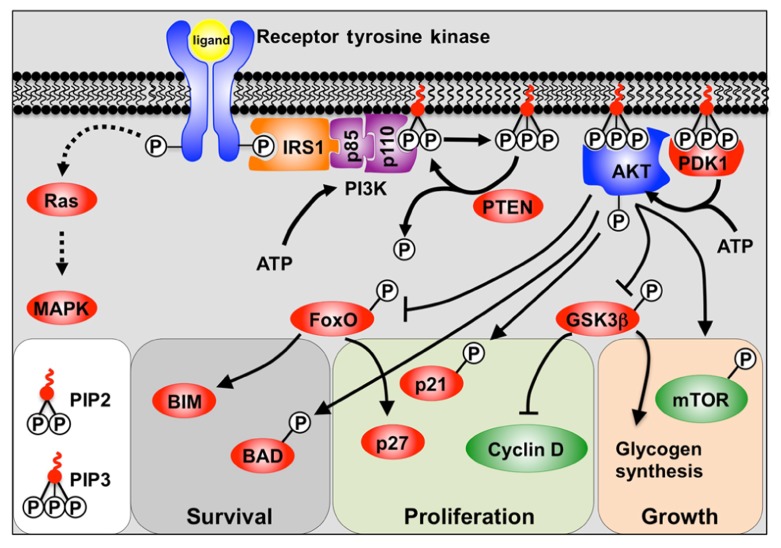
**A model depicting PI3K signaling**. Binding of the receptor tyrosine kinase ligand activates receptor auto-phosphorylation, leading to recruitment of substrate proteins such as IRS-1. This leads to recruitment of the regulatory (p85) and catalytic (p110) subunits of class 1a PI3K. PI3K phosphorylation of PIP_2_ to PIP_3_ allows PIP_3_ to act as a secondary messenger within the inner surface of the cell membrane. AKT and PDK1 bind to PIP3, and PDK1 and mTOR/Rictor activate AKT via phosphorylation. Active AKT is then able to promote cell survival, growth, and proliferation by phosphorylation of key substrates. Also shown is the alternative Ras pathway which can also be stimulated by receptor tyrosine kinases to activate MAPKs.

The PI3K pathway is activated through the receptor tyrosine kinase (RTK) class of receptors which include fibroblast growth factor receptors (FGFRs), insulin-like growth factor receptors (IGFRs), and insulin receptor (**Figure 1**). Engagement of the ligand with the RTK causes autophosphorylation of tyrosine residues in the cytoplasmic domain. These phosphorylated tyrosine residues then recruit docking proteins, most notably IRS1, which in turn recruits the p85 subunit of PI3K. PI3K itself consists of a regulatory p85 subunit and a catalytic p110 subunit. Recruitment of the PI3K complex to the inner surface of the plasma membrane juxtaposes it with its substrate phosphatidylinositol-4,5 diphosphate (PIP_2_) located in the inner lamina of the cell membrane. PIP_2_ is then phosphorylated by the p110 subunit to form phosphatidylinositol-3,4,5 trisphosphate (PIP_3_). The conversion of PIP_3_ to PIP_2_, and the subsequent inactivation of PI3K downstream signaling, is facilitated by the tumor suppressor and phosphatase; phosphatase and tensin homolog deleted on chromosome ten (PTEN) (Cantley and Neel, 1999).

Phosphatidylinositol-3,4,5 trisphosphate then acts as a secondary messenger within the membrane, recruiting the binding of proteins containing pleckstrin homology (PH) domains to the inner surface of the cell membrane. Most notable amongst the PH domain containing proteins are AKT (also known as protein kinase B, PKB) and PDK1. The AKT/PKB family is comprised of three separate genes (*AKT1–3*) and all encode serine/threonine protein kinases. Upon translocation to the cell membrane, AKT is phosphorylated by PDK1, and the mTORC2/Rictor complex, which provides full activation of AKT (Cantley and Neel, 1999).

Whilst RTKs activate AKT via PI3K, they can also activate mitogen-activated protein kinases (MAPKs) such as ERK1/2 via the Ras/Raf pathway (Ramos, 2008).

## AKT TARGETS

AKT activation is generally associated with the processes of cell survival, growth, and proliferation (Manning and Cantley, 2007). Cell survival is chiefly regulated by inhibitory phosphorylation of the FoxO family of transcription factors by AKT, and thus attenuation of their pro-apoptotic target gene *Bim* (Dijkers et al., 2002; Sunters et al., 2003), as well as by direct inactivation by phosphorylation of Bad (Datta et al., 2002). In contrast, cell growth is regulated by activation of mTOR (mammalian Target of Rapamycin) thereby promoting protein synthesis (Wullschleger et al., 2006). Proliferation is stimulated via a number of mechanisms including inhibition of the FoxO-mediated transcription of the cyclin-dependent kinase inhibitor p27^Kip1^ (Dijkers et al., 2000; Ho et al., 2008). AKT also inhibits both p27^Kip1^ and p21^Cip1^ function by phosphorylation (Zhou et al., 2001; Liang et al., 2002), as well as by regulating D-type cyclins (Liang and Slingerland, 2003).

## OSTEOBLAST DIFFERENTIATION

Osteoblast differentiation begins with MSC commitment to the osteoblast lineage by expression of the osteoblast-specific transcription factor Runx2 (Otto et al., 1997). New osteoprogenitors proliferate then express the matrix promoting proteins: collagen 1a (Col-1a; Bellows et al., 1986; Lee et al., 2000), osteopontin (OPN; Hay et al., 1999; El-Tanani et al., 2004), alkaline phosphatase (ALP; Aronow et al., 1990), and bone morphogenic proteins (BMPs; van der Horst et al., 2002; Rosen, 2006). Integrin activation by the newly synthesized matrix promotes maturation into pre-osteoblasts, which express the transcription factor Osterix (Osx; Xiao et al., 1998; Meyers et al., 2004). As pre-osteoblasts mature, Runx2 and Col-1a expression declines, whilst Bone Sialoprotein (Chen et al., 1997; Lai and Cheng, 2005) and Osteocalcin expression increases (Hay et al., 1999).

## TOO MUCH PI3K/AKT – “JUST BIG BONED?”

One of the problems in delineating a truly osteoblastic role for PI3K/AKT signaling in bone biology is the dissection of the generic effects (survival, proliferation, and growth) from those that are limited to osteoblasts. However, genetic studies using transgenic and knockout mice targeting the PI3K/AKT pathway have yielded informative results. Original global deletions of the *PTEN *tumor suppressor resulted in cells with elevated PI3K/AKT signaling, but embryonic lethality (Di Cristofano et al., 1998; Suzuki et al., 1998). However, when mice containing *Cre* under the control of the *Col2a1* promoter were crossed with mice containing a floxed *PTEN *allele, *PTEN* was knocked out in osteo-chondroprogenitor cells (Ford-Hutchinson et al., 2007). These mice exhibited a disorganized growth plate, excessive matrix production, and elevated AKT and S6K activation in growth plate chondrocytes and osteoblasts of the primary spongiosa (Ford-Hutchinson et al., 2007). The mice developed elongated long bones containing extensive trabeculation and increased cortical thickness, but interestingly no increase in chondrocyte proliferation (Ford-Hutchinson et al., 2007). Guntur et al. (2011) generated mice in which *PTEN *was ablated solely in osteoprogenitors. The osteoprogenitors in these mice had increased proliferation and survival, and this expanded pool of progenitors differentiated rapidly. However, accelerated osteoblast differentiation caused precocious osteoid deposition in the developing perichondrium, which was uncoupled from interaction with chondrocytes, resulting in shorter but broader bones (Guntur et al., 2011). Late stage osteoblast-specific *PTEN* knockout mice were obtained by crossing *osteocalcin*-*Cre* with floxed *PTEN *mice. These mice were of usual size, but exhibited increased bone formation throughout life due to elevated AKT-mediated cell survival (Liu et al., 2007). Whilst the PTEN knockout mice have hinted the consequence of elevated PI3K signaling to AKT, it is only recent studies using AKT knockout mice that have provided more detailed information regarding isoform specificity in osteoblast biology.

## NOT ENOUGH PI3K/AKT – “SMALL FRY”

Global *AKT-1* knockout mice are small with reduced bone mineral density, suggesting a potential osteoblast-specific effect (Kawamura et al., 2007). However, this may be a more generic effect, and indeed was attributed to an increase in apoptosis rates mediated by a failure of AKT1 to repress FoxO3a transcription of *Bim* (Kawamura et al., 2007). More specifically a reduction in Runx2 target gene expression was observed, including RANKL, which resulted in decreased osteoblast induced osteoclastogenesis and slow bone remodeling (Kawamura et al., 2007). Global *AKT-2* knockouts develop severe diabetes, but skeletally they only display a very mild decrease in weight and length (Garofalo et al., 2003). *AKT-1/AKT-2* double knockouts die shortly after birth and exhibit dwarfism (Peng et al., 2003). The osteoblastic phenotype is extreme, with embryos having negligible ossification (Peng et al., 2003) suggesting some functional redundancy between AKT1 and 2. *AKT3* knockout mice are viable, have reduced brain size, but no discernable skeletal phenotype (Easton et al., 2005). Taken together, these genetic models suggest a specific role in bone is confined to AKT1 and/or 2, and occurs both in an osteoblast cell autonomous manner and via their interactions with chondrocytes and osteoclasts.

## STIMULATING PI3K/AKT IN BONE

The *AKT* and *PTEN* knockouts indicate that skeletal development and functional integrity rely on fine-tuning of the AKT signaling pathway, itself controlled by other signaling molecules, the most important of which are fibroblast growth factor (FGF) and insulin-like growth factor (IGF).

The FGF family consists of at least 22 members coupled with at least 5 receptors (FGFRs) (Bottcher and Niehrs, 2005; Coutu and Galipeau, 2011). During endochondral ossification FGFR3 is the predominant receptor expressed by condensing mesenchymal chondrocytes and proliferating chondrocytes in the growth plate, whilst FGFR1 is expressed by hypertrophic chondrocytes, and thought to play a role in blocking proliferation. In osteoblasts FGFR-1 and -2 are both expressed, and FGF2 stimulates the proliferation of osteoblast precursors (Yu et al., 2003).

IGF-1 plays a crucial role in postnatal bone growth, especially during puberty. The majority of circulating IGF-1 is produced by the liver (DiGirolamo et al., 2007; Yakar et al., 2010), and mice with a conditional *IGF-1 *knockout in the liver are essentially normal, but have reduced circulating IGF-1 and a reduction in cortical bone deposition (Yakar et al., 2009). However, mice with a targeted *IGF-1R* deletion in osteoblasts exhibited a time lag between osteoid deposition and mineralization, and thus make poor quality bone (Zhang et al., 2002). Conversely, osteoblast-specific *IGF-1* overexpression in mice increased trabecular bone volume without any associated hyperplasia (Zhao et al., 2000). These findings demonstrate that local IGF-1 signaling also influences bone architecture and mineralization. Insulin, like IGF-1, has been shown to be anabolic in bone, and induces osteocalcin expression, but lacks the mitogenic effects of IGF-1 (Zhang et al., 2012).

Whilst IGF and FGF signaling activates AKT via PI3K, they also have the ability to activate MAPKs (Ramos, 2008), and the relative contributions of these two pathways to osteoblast function remain unclear. Whilst the osteopenia observed in osteoblast-specific Gab1 knockout mice has been linked to impaired IGF-1/insulin signaling via both PI3K and Ras pathways (Weng et al., 2010), recent data suggests the mitogenic effect of IGF-1 requires both pathways, whilst differentiation relies on PI3K/AKT signaling (Raucci et al., 2008).

## INTERACTION BETWEEN PI3K/AKT AND OSTEOGENIC FACTORS

Recent evidence suggests that Runx2 functions more as a promoter organizer rather than a transcription factor, acting as a central hub to recruit transcriptional co-activators such as CBP (Schroeder et al., 2005) or inhibitors such as Sin3/histone deacetylases (Westendorf, 2006). Furthermore, Runx2 interacts with other transcription factors such as AP-1 and estrogen receptor alpha (ERα) (Westendorf, 2006; Khalid et al., 2008; Chen et al., 2012). The ability of Runx2 to function as a transcriptional activator or repressor is fine-tuned by phosphorylation, for example, ERK1/2 and p38-MAPK phosphorylation promote osteoblast differentiation (Xiao et al., 2002; Greenblatt et al., 2010). In contrast, JNK1 phosphorylation of Runx2 is inhibitory, blocking the early stages of differentiation (Huang et al., 2012), however, since JNK activity is associated with the terminal stages of differentiation, this inhibition of Runx2 might correlate with reduced Runx2 function in mature osteoblasts (Matsuguchi et al., 2009).

Although Runx2 target gene expression is reduced in *AKT-1* knockout mice, Runx2–PI3K/AKT interactions are unlikely to occur via direct phosphorylation because Runx2 lacks an AKT consensus phosphorylation site (Kawamura et al., 2007). Notwithstanding, direct phosphorylation by AKT blocks the ability of GSK3β to inhibit Runx2 DNA binding (Kugimiya et al., 2007) and of FoxO1 to represses Runx2-dependent osteocalcin transcription (Yang et al., 2010; Zhang et al., 2011). A potential feed-forward loop between Runx2 and PI3K/AKT may also exist as Runx2 activates p85 and p110β PI3K subunit transcription (Fujita et al., 2004).

Osterix is a Runx2 target gene that stimulates osteoblast lineage commitment and promotes osteoblast maturation (Komori, 2006; Nishio et al., 2006; Zhou et al., 2010). Apart from the effects of PI3K/AKT on Runx2, there is little evidence of direct phosphorylation of Osx by AKT (Choi et al., 2011), but PI3K/AKT is required for BMP-induced Osx transcriptional activity (Mandal et al., 2010; Choi et al., 2011).

## OSTEOGENIC SIGNALING PATHWAYS

### CANONICAL WNT SIGNALING

During absence of Wnt ligands, the kinase GSK3β phosphorylates the transcriptional co-activator β-catenin, thereby targeting it for proteosomal degradation. Engagement of Wnt ligands with the Frizzled/Lrp5 or -6 co-receptor inhibits GSK3β, leading to the accumulation of hypo-phosphorylated β-catenin, which translocates to the nucleus in order to stimulate Lef/TCF target gene expression. Wnt signaling functions during skeletal development partly by promoting osteoblastic commitment (Hill et al., 2005). Humans with inactivating or activating mutations in Lrp5 exhibit low or high bone mass, respectively, implicating Wnt in postnatal bone homeostasis (Whyte et al., 2004; Ai et al., 2005; Ferrari et al., 2005). Inhibitory phosphorylation of GSK3β by AKT results in the activation of β-catenin (Smith and Frenkel, 2005; Sunters et al., 2010). Furthermore, direct phosphorylation of β-catenin by AKT has been shown to increase its capacity for transcriptional activation of Lef/TCF target genes (Fang et al., 2007).

### BMP SIGNALING

Bone morphogenic proteins are members of the TGFβ family and bind to tetrameric type I and II receptors on the cell surface which phosphorylate members of the SMAD family (SMAD 1, 5, and 8). Phospho-SMADs bind to SMAD4 and translocate to the nucleus to regulate gene expression. Blocking BMP signaling with the BMP antagonist Noggin reduces osteoblast differentiation, and results in mice with severe osteoporosis (Wu et al., 2003). Repression of BMP signaling by Twist 1/2 prevents osteoblast commitment by mesenchymal precursors by silencing Runx2, AP, and OPN transcription (Bialek et al., 2004; Hayashi et al., 2007). Mice lacking BMP2 and -4 in limb bud mesenchyme have impaired osteogenesis (Bandyopadhyay et al., 2006), and whilst *BMP2* knockout mice do make bone, they exhibit a mineralization deficit making them susceptible to fractures (Tsuji et al., 2006). BMP3 is produced by mature osteoblasts and osteocytes to prevent osteoblast differentiation, providing a negative feedback loop to control osteoblast numbers (Kokabu et al., 2012). PI3K/AKT is required for BMP-induced Osx activation (Osyczka and Leboy, 2005; Mukherjee and Rotwein, 2009; Mandal et al., 2010; Choi et al., 2011), this dependency would also appear to be isoform specific, since recent work by Mukherjee et al. (2010)**demonstrated that a unique function of AKT2 is required for BMP2-mediated osteoblast differentiation.

AKT has been also shown to be activated by BMPs via a mechanism that is not fully understood (Osyczka and Leboy, 2005; Mukherjee and Rotwein, 2009; Mukherjee et al., 2010). Intriguingly, osteogenic BMP targets include the Id family of genes which can inhibit the Twist blockade of BMP-induced transcription, and thus osteogenesis (Ogata et al., 1993; Miyazono and Miyazawa, 2002; Ying et al., 2003; Peng et al., 2004; Nakashima et al., 2005). Id1 transcription is repressed by FoxOs (Birkenkamp et al., 2007), and it is possible that PI3K/AKT inactivation of FoxOs could cooperate with SMADs in the BMP-mediated activation of Id1. Furthermore, BMP and Id1 can activate AKT via PTEN repression (Beck and Carethers, 2007; Chow et al., 2007, 2008; Lee et al., 2009), however, this is unlikely to be the only mechanism responsible. It is attractive to speculate that these findings may represent reciprocal activation between PI3K/AKT and BMP signaling – which could potentially amplify osteogenic responses to BMP, IGF-1, and FGFs.

## PATHOLOGIES INVOLVING PI3K/AKT

The PI3K/AKT signaling pathway in osteoblasts is clearly important for normal skeletal development and homeostasis, however, it is also implicated in various pathological conditions.

### OSTEOPOROSIS AND MECHANICAL STRAIN

Bone loss and fracture susceptibility that characterize osteoporosis are often thought of as symptomatic of an attenuation of resident bone cells’ ability to use the mechanical strain engendered by normal load bearing activity as a stimulus to remodel both bone mass and architecture (Lanyon, 2008; Price et al., 2010). The initial detection of strain is thought to occur in osteocytes (Bonewald, 2007), but does also occur in osteoblasts, and involves signaling via ERα (Jessop et al., 2002; Lee et al., 2003; Zaman et al., 2010), production of prostaglandins (Zaman et al., 1997), nitric oxide (Pitsillides et al., 1995), ATP (Rumney et al., 2012), canonical Wnt signaling (Lau et al., 2006; Armstrong et al., 2007), IGFs (Zaman et al., 1997; Cheng et al., 1999), and the suppression of the soluble Wnt antagonist sclerostin (Galea et al., 2011). With regard to PI3K/AKT signaling, activation of β-catenin by strain requires AKT-mediated inhibition of GSK3β (Case et al., 2008; Sunters et al., 2010), which also occurs in osteocytes (Kitase et al., 2010). AKT activation occurs via PI3K and is Wnt independent, but depends on the formation of a complex between ERα and IGF-1R which increased the responsiveness of IGF-1R to ambient levels of IGF-1 (Sunters et al., 2010). Whilst administration of IGF-1 to osteoporosis patients has little positive effect on bone density (Friedlander et al., 2001), osteoblasts isolated from osteoporotic donors have an attenuated PI3K/AKT response to IGF-1, suggesting that IGF-1R responsiveness may play a role in osteoporosis (Perrini et al., 2008).

### OSTEOSARCOMA

Osteosarcoma is the most common malignancy affecting the skeleton, and although relatively rare in humans (Broadhead et al., 2011), is much more common in dogs, especially large and giant breeds (Tjalma, 1966; Chun and de Lorimier, 2003; Chun, 2005). Since susceptible large breed dogs have higher IGF-1 levels (Burrow et al., 1998), and IGF-1R is often overexpressed in canine osteosarcomas (Eigenmann et al., 1984; Sutter et al., 2007), a link between IGF-1 signaling and osteosarcoma has been proposed. In support of this, PTEN is commonly down regulated, mutated, or deleted in many canine and human osteosarcomas (Levine et al., 2002; MacEwen et al., 2004) and is associated with elevated AKT activation, suggesting that PI3K/AKT may play a causative role in osteosarcoma formation.

It is possible that other conditions associated with increased or decreased bone formation may have perturbed PI3K/AKT function as a common event. For example, the PTEN bone-specific knockout mouse, which results in an increase in AKT signaling, has increased bone formation, leading to osteopetrosis (Liu et al., 2007). Moreover, mutations in Irs1 resulting in reduced AKT phosphorylation lead to reduced bone formation (DeMambro et al., 2010). However, effects need not be manifest globally in the whole skeleton as demonstrated by the Twist haploinsufficiency model, where down regulation of the ubiquitin ligase Cbl promotes AKT signaling through a reduction in PI3K degradation. The result is increased bone formation specifically in the coronal suture in Saethre-Chotzen syndrome (Guenou et al., 2006).

## SUMMARY

Given the generic roles of the PI3K/AKT pathway, it is not unexpected that it would play a role in osteoblasts. However recent evidence suggests that some of these generic effects are *selectively* activated in osteoblasts during normal physiology. Additional specificity is introduced when one considerers the highly selective downstream interactions between the PI3K/AKT pathway and other pathways controlling osteoblast differentiation and function. Our contention is that the PI3K/AKT pathway may well be a central nexus in the networks of signaling pathways that helps to fine-tune osteoblast differentiation and homeostasis to produce a normal skeleton. Thus AKT represents a viable therapeutic target in multiple skeletal diseases.

## Conflict of Interest Statement

The authors declare that the research was conducted in the absence of any commercial or financial relationships that could be construed as a potential conflict of interest.
